# Erythrocyte depletion from bone marrow: performance evaluation after 50 clinical-scale depletions with Spectra Optia BMC

**DOI:** 10.1186/s12967-017-1277-6

**Published:** 2017-08-11

**Authors:** Soo-Zin Kim-Wanner, Gesine Bug, Juliane Steinmann, Salem Ajib, Nadine Sorg, Carolin Poppe, Milica Bunos, Eva Wingenfeld, Christiane Hümmer, Beate Luxembourg, Erhard Seifried, Halvard Bonig

**Affiliations:** 1 Institute Frankfurt, German Red Cross Blood Service Baden-Württemberg-Hessen, Sandhofstr. 1, 60528 Frankfurt, Germany; 2 Division of Stem Cell Transplantation, Department of Medicine II, Goethe University, Frankfurt, Germany; 3 Department of Hemostaseology, Deutsche Klinik für Diagnostik, Wiesbaden, Germany; 4 Institute for Transfusion Medicine and Immunohematology, Goethe University Medical Center, Sandhofstr. 1, 60528 Frankfurt, Germany; 5 Department of Medicine/Hematology, University of Washington, Seattle, WA USA

**Keywords:** Cell processing, RBC-depletion, Apheresis

## Abstract

**Background:**

Red blood cell (RBC) depletion is a standard graft manipulation technique for ABO-incompatible bone marrow (BM) transplants. The BM processing module for Spectra Optia, “BMC”, was previously introduced. We here report the largest series to date of routine quality data after performing 50 clinical-scale RBC-depletions.

**Methods:**

Fifty successive RBC-depletions from autologous (n = 5) and allogeneic (n = 45) BM transplants were performed with the Spectra Optia BMC apheresis suite. Product quality was assessed before and after processing for volume, RBC and leukocyte content; RBC-depletion and stem cell (CD34+ cells) recovery was calculated there from. Clinical engraftment data were collected from 26/45 allogeneic recipients.

**Results:**

Median RBC removal was 98.2% (range 90.8–99.1%), median CD34+ cell recovery was 93.6%, minimum recovery being 72%, total product volume was reduced to 7.5% (range 4.7–23.0%). Products engrafted with expected probability and kinetics. Performance indicators were stable over time.

**Discussion:**

Spectra Optia BMC is a robust and efficient technology for RBC-depletion and volume reduction of BM, providing near-complete RBC removal and excellent CD34+ cell recovery.

## Background

Considering the independent inheritance of MHC and ABO and the latter’s distribution in the general population, roughly one in five allogeneic BM transplantations will be from ABO major or bi-directionally mismatched donors [1]. Given the current preference for PBSC over BM grafts, nevertheless only approximately 600 ABO major/bi-directionally mismatched BM transplants are currently performed per year in Europe. With more than 600 allogeneic transplant programs in Europe [2], this leaves one of these per year per center on average, clearly too little for each to develop, optimize and validate RBC-depletion protocols for BM grafts. That said, changing clinical trends, namely transplant protocols using unmanipulated haplo-identical BM followed by intermediate-dose cyclophosphamide to deplete allo-reactive T-cells in vivo [3], are leading to a renaissance of BM as transplant source and hence, are expected to increase the frequency of RBC-depletions. Therefore, pre-validated protocols on readily available technology platforms with as high a degree of automation as possible are required to satisfy these many centers’ need for a robust process that is as sporadically used as its outcome is vitally critical to the success of the transplantation.

We here present data suggesting that Spectra Optia BMC may fit that description. It is the most recently developed application for the Spectra Optia apheresis device [4–12] which was previously introduced with respect to feasibility and initial performance data [13, 14]. Results indicating high reproducibility and predictability of outcomes as well as clinically adequate depletion efficiency and transplant function in routine clinical use are summarized for 50 successive clinical-scale RBC-depletions performed in an academic GMP setting over the last 4 years.

## Methods

### Patients, donors and ethical considerations

BM was aspirated under general anesthesia from healthy registry or family donors (n = 45) as allogeneic transplants, or from patients (n = 5) with non-malignant illnesses providing an indication for allogeneic stem cell transplantation, to serve as cryopreserved autologous back-ups because of these patients’ inherently high risk of allogeneic graft rejection. BM was anti-coagulated with ACD-A and Heparin as per local protocol. Both locally collected BM products and products from cooperating collection sites elsewhere were eligible for RBC depletion at our center. RBC depletions were mostly performed for the local allogeneic transplant services at Goethe University Hospital, Frankfurt (pediatric and adult) and Deutsche Klinik für Diagnostik, Wiesbaden (adult), as well as for several other collaborating transplant programs in Germany. Allogeneic donor assessment was done according to WMDA recommendations as reported [15, 16]. This being an anonymized retrospective analysis of routine clinical data, no specific ethics approval was required.

### Graft quality assessment

Product quality including hematocrit (Hct) was assessed by automatic hemocytometry (Sysmex XT-1800i hematology analyzer, Sysmex, Norderstedt, Germany). CD34+ cells were enumerated using the single-platform BD SCE kit and FACSCalibur flow cytometer (Becton–Dickinson, Heidelberg, Germany), as described [17]. Volume was assessed by product weight with correction for hematocrit.

### Separation device and protocol

The semi-automatic RBC-depletion protocol using the Spectra Optia separator with the BMC software, the standard Spectra Optia MNC filler, standard Spectra Optia IDL tubing set and the BMC accessory kit, a large BM bag for re-circulating of the (increasingly MNC-depleted) BM suspension were previously introduced [14]. Spectra Optia fully automatically performs photo detector-guided apheresis of the BM, physically highly analogously to MNC apheresis in stem cell donors. In addition, interphase and color of collection flow are monitored visually by technicians and “collection preference” is adjusted as needed, as well as the BM bag is gently agitated every few minutes.

The set was installed and primed as directed by the manufacturer. BM is processed without further anticoagulation; therefore, the anticoagulant line of the apheresis kit was sealed during set installation. An initial collection preference [4] of 50 was selected, as manufacturer recommended; RBC-depletions were run in the automatic mode. Hematocrit was tracked using the colorgram from the COBE Spectra device, to maintain an apparent hematocrit in the collection line of approximately 5%; adjustment of the collection preference to 30–35 was generally required to warrant that. Once established, the interphase was stable and very few changes in collection preference had to be undertaken. The entire BM product volume was processed 8–9 times with a typical flow velocity of 120 mL/min and the centrifuge at full speed, which yields a packing factor of approximately 10.

### Engraftment analyses

Post-transplant follow-up was received of the 26 allogeneic recipients treated at Goethe University Hospital; data comprising day with ANC >500/µL (neutrophil engraftment), day with platelet count >50/nL without transfusion (platelet engraftment), or day with hemoglobin >8 g/dL without transfusion (estimate of RBC engraftment) were analyzed according to donor histocompatibility, conditioning regimen and major vs. minor ABO mismatch.

### Data analysis

RBC volume (mL) was calculated from hematocrit and product volume. RBC depletion (%) was calculated as the quotient of pre- and post-process RBC volume. CD34+ cell recovery (%) and volume reduction (%) were similarly calculated from pre- and post-processing total CD34+ cell dose or volumes. The Shapiro–Wilk test was used to test parameters for normality. Differences between groups were calculated with Mann–Whitney U test or H test of Kruskal and Wallis, as appropriate. Potential changes in performance indicators over time were calculated by stratifying depletion data by year. Values are given as median and range. A p value of <0.05 was considered significant. Engraftment data are indicated as median day of engraftment for each of the lineages. Statistical analyses were performed with the SPSS software package, version 24.0.

## Results

### Feasibility

**Fig. 1:**
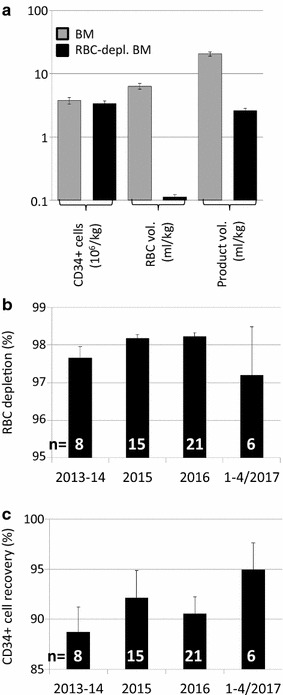
RBC depletion from BM. RBC depletions from BM were performed with Spectra Optia BMC. **a** CD34+ cell dose (10^6^/kg), RBC dose (mL/kg), and product volume (mL/kg) in unmanipulated BM (*grey*) and RBC depleted BM (*black*) are shown (mean ± SEM). **b** Efficiency of RBC depletion over time as % of RBC volume in RBC depleted vs. starting product. **c** Efficiency of CD34+ cell recovery over time as number of CD34+ cells in RBC depleted vs. starting product, expressed as %

50 successive Spectra Optia BMC processes proceeded uneventfully and successfully, in that they yielded RBC-depleted BM transplants in agreement with the pre-defined specification of the licensed product (marketing authorization PEI.G.03647.02.1). The processed BM samples were all clinical transplants which were either transplanted fresh (all allogeneic products, n = 45) or cryopreserved (all autologous products, n = 5, none of which was used clinically to date). The BM samples in this series were sized between 414 and 2045 mL (median 1282 mL) including anti-coagulants. Spectra Optia removed 98.2% (range 90.8–99.1%) of RBCs while retaining 93.6% of CD34+ cells (range 72–100%), yielding products of 56–327 mL (range; median 167.5 mL) (Fig. 1a). Post-processing WBC viability was 96.1% (range 80.0–99.0%). Apheresis, due to the differential density of granulocytes and MNC, recovers MNC with much higher efficiency, so that total WBC recovery was 62% (34–85%). T-cell recovery was not directly assessable since T-cell content in starting material was not enumerated, but post-RBC-depletion, there were 12-fold (range 6–18) more T-cells than HSC, in agreement with typical ratios for BM.

**Fig. 2:**
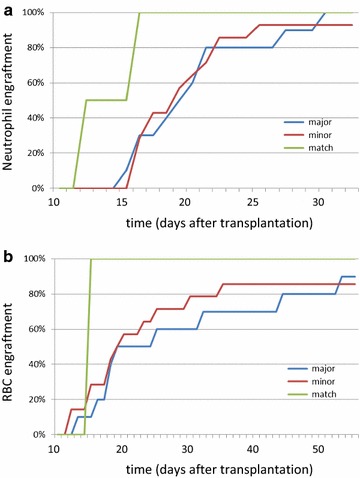
Engraftment data for RBC depleted BM aspirates. **a** Engraftment for neutrophils was analyzed based on ABO match. Probability of engraftment over time is shown. Median time to engraftment was no different for the groups. **b** Engraftment for RBCs was analyzed based on ABO match. Probability of engraftment over time is shown. Median time to engraftment was no different for the groups (n for ABO matched/minor/major mismatch: 2/14/10)

**Fig. 3:**
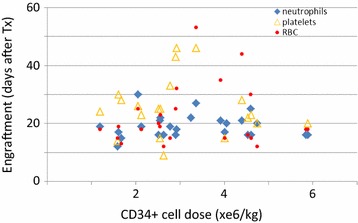
Effect of CD34+ cell dose in RBC-depleted allogeneic BM products on engraftment velocity for different hematopoietic lineages. Engraftment for neutrophils (*diamonds*), platelets (*triangles*) and RBCs (*circles*) for Spectra Optia BMP is plotted by CD34+ cell dose (×10^6^/kg; X-axis) over time (days post-transplant; Y-axis). RBC depleted allogeneic BM products provided timely engraftment in all lineages. A dose–effect was not apparent

Engraftment reports were obtained for 26 recipients of allogeneic RBC depleted BM grafts. 12 had acute myeloid leukemia (AML) or secondary AML, 4 aplastic anemia, 3 each acute lymphoblastic leukemia (ALL) or chronic myeloid leukemia (CML), 2 myelodysplastic syndrome (MDS) and 1 each primary myelofibrosis (PMF) and Hodgkin’s disease (HD). 19, 4 or 3 patients received myeloablative, reduced-intensity or mini-conditioning, respectively, followed by transplantation of grafts from identical (3) or haplo-identical (14) family donors or matched (7) or mismatched (2) unrelated donors. With respect to ABO matching, for 10 products the indication for RBC-depletion was ABO major mismatch, for 14 ABO minor mismatch. In these, the per-kg dose of CD34+ cells was 1.2–5.9 × 10^6^ (range; median 2.9 × 10^6^). Overall median engraftment for neutrophils (ANC >500/µL), platelets (spontaneous plt >50/nL) and RBCs (spontaneous Hgb >8.0 g/dL) was 18/25/18 days and hence, within expectation. One patient with neutrophil engraftment on day 20 died of septicemia 2 days thereafter without achieving RBC and platelet engraftment. Specifically, median engraftment for neutrophils, platelets and RBCs was 16/28/18 vs. 19.5/24/19 days for haplo vs. better MHC match, 18/28/18 vs. 19.5/22.5/20.5 days for MAC vs. non-MAC conditioning, and 19/28/19 vs. 18/25.5/18.5 vs. 14/18.5/15 days for ABO-major mismatch vs. ABO-minor mismatch vs. ABO-match (Fig. 2) (p = n.s. for all comparisons, but n for ABO-matched transplantations was only 2). We also assessed potential effects of CD34+ cell dose on engraftment velocity; engraftment was achieved in a timely manner independent of CD34+ cell dose over the entire dose range administered in this series, including at the lowest CD34+ cell doses (1.2 × 10^6^/kg) (Fig. 3). No data for GvHD were reported.

## Discussion

A typical dose of BM cells for transplantation is 2.5–3.0 × 10^8^/kg nucleated cells [18–20], equivalent to approximately 12–20 mL BM aspirate/kg. At an average hematocrit of 30–35%, the RBC content of BM aspirate is thus equivalent to 2–3 RBC concentrates and hence not unconditionally tolerable for ABO major mismatched recipients. One long-established method to avoid severe and potentially lethal intravascular immune hemolysis during BM infusion is RBC depletion from the graft [21]. An average BM transplant also contains plasma in quantities equivalent to three units of plasma, of potential clinical relevance in patient–donor ABO minor incompatibility constellations, and the RBC depletion process is thus also used for plasma reduction in these cases, as well as RBC depletion is used for volume reduction of BM aspirate, e.g. prior to cryopreservation, which mostly applies to autologous BM, or for very small pediatric transplant patients.

Differences in size and density between RBCs, WBCs, platelets and plasma allows for their efficient separation solely by density gradient centrifugation or apheresis. Both methods have been applied successfully for clinical transplant manufacturing [21–24]. The critical quality-defining parameters for the success of RBC-depletion of BM are recovery of CD34+ “stem cells” which are contained within the mononuclear cell population and, when performed in the context of ABO-major-incompatible BM-transplantation, near-qualitative removal of RBCs to values typically observed in peripheral blood stem cell apheresis products, i.e. to values of ≤0.5 mL/kg body weight of the recipient.

Apheresis technology has been successfully used to deplete RBCs from BM, for instance with COBE Spectra and its successor, Spectra Optia BMC, which was first introduced 4 years ago [14]. One of the possibly most obvious advantages of Spectra Optia BMC over its potential competitors, including COBE2991 [21, 25–27], Sepax II NeatCell [28–30] or CliniMACS Prodigy [14], is the fact that it can be used for many other clinical apheresis applications, so that depreciation and maintenance can be distributed over a large number of processes, compared to devices designated only for RBC depletion from BM (and other RBC-replete products) which are burdened with much higher pro-rated costs. Moreover, the relatively greater frequency of peripheral blood apheresis procedures guarantees a certain familiarity of the operators with the apheresis machine which in most centers cannot be achieved with designated RBC-depletion technologies, given their relative rarity. The technology can accommodate large BM volumes, for which reason it is used as the standard technology in our center. The high minimal RBC content required to raise the interphase in the connector, 125 mL, can be an obstacle when dealing with low-volume pediatric BM products.

As we are showing, Spectra Optia BMC is a robust and efficient technology for RBC depletion from BM which was flawlessly effective in all 50 sequential preparations. Residual RBC volumes were no larger than 9.2 mL total (≤0.4 mL/kg) (median 1.8 mL total/0.1 mL/kg), thus in all cases below the specified maximal volume of 0.5 mL/kg. RBC reduction achieved with Spectra Optia BMC is less complete than when density gradient centrifugation is performed [14], but clinically entirely sufficient. The recovery of CD34+ cells, the most important active ingredient of “stem cell” transplants exceeded 72% in all cases (median 93.6%); together with the engraftment data, which confirm previous analyses of ours negating strong dose effects for stem cell numbers in allogeneic BM grafts in the range that is typically clinically administered, our data indicate adequate stem cell recovery. Functionality of RBC-depleted BM as stem cell graft is demonstrated by engraftment data which for all examined lineages are in line with expectations. RBC depletion and CD34 recovery outcomes for Spectra Optia BMC were in a similar range as those reported for the predecessor technology, COBE Spectra, and for the initial performance reports on Spectra Optia published previously [22, 31, 32].

Alternatives to RBC depletion, such as in vivo isoagglutinin depletion have in the last years been developed [33], in part because of concern about CD34+ cell loss during BM processing. Our data indicate that this concern is not justified.

With many new technologies, even partly automated ones, learning curves are observed. Thus when Spectra Optia MNC was first introduced, marked improvements of apheresis yields were noted over the first couple of 100 stem cell apheresis [4, 5]. Given that most centers will perform very few RBC depletions, we analyzed the quality of RBC-depletion outcomes over time for potential evidence of a “learning curve”. Since already the very first RBC depletions with the new technology had been uneventful and quite satisfactory in quality, the room for improvement was modest. And indeed despite performance measures trending slightly upwards over time no statistically significant (let alone clinically relevant) improvement over time was observed, neither in total quantitative measures for CD34+ cell recovery or RBC-depletion, nor in the spread, or predictability, of outcomes, when comparing year-by-year outcomes from 2013/2014 RBC depletions up to 2017 (p = 0.341 for RBC depletion and p = 0.437 for CD34+ cell recovery) (Fig. 1b, c). These data raise the expectation that centers planning to adopt RBC depletion with Spectra Optia will also not require significant practice with surrogate materials before they can expect to achieve clinically useable RBC depletions of BM.

The paucity of BM transplants in need of RBC depletion and the difficulty of obtaining large-volume volunteer BMs for experimentation does not allow for thorough optimization and validation of BM RBC depletion. It is clear that changes to any of the many adjustable apheresis variables on Spectra Optia BMC can have significant effects on product properties. However, we operated with default settings for inlet flow, packing factor and collection flow and only adjusted collection preference, so that we would collect a product with a hematocrit of approximately 5% (residual RBC content of <10 mL). With these settings, Spectra Optia performed quite satisfactorily. While theoretically products with even lower RBC content can be collected with Optia [4–6, 8], this was not attempted: the effort seemed unnecessary because a satisfactory RBC depletion was already achieved with default settings, while a lighter product color would increase the risk of inferior target cell recovery. Similarly, packing factor was not varied from default settings; a higher one might have resulted in a crisper interphase and lighter product, a lower one might have allowed for more efficient platelet reduction. We previously documented that expected consequences of variations of apheresis protocols are not always observed, cautioning against overly courageous changes in apheresis variables [5, 8]. Our data document feasibility and adequate efficiency of RBC-depletion while recovering most target cells with Optia when using default settings and only adjusting collection preference (collection line “color”).

The capacity for handling large BM volumes, very satisfactory (functional) product properties, but also speed and simplicity/robustness of the method due to the high degree of automation and the acceptable costs of Spectra Optia BMC suggest its use for the typical BM product for hematopoietic reconstitution, the volume of which exceeds 300 mL. Availability of a second technology may be desirable for processing of smaller volumes of BM such as would be collected from or for light-weight pediatric donors or from patients for regenerative medicine purposes [29] because Spectra Optia BMC requires a minimal RBC volume of 125 mL. Also for RBC-depletion of less strongly RBC-contaminated products which nevertheless require further RBC reduction, e.g. poorly collected peripheral blood stem cell products, Ficoll-based technologies could be very useful. However, as we recently demonstrated, such products can alternatively be spiked with donor-compatible RBCs to raise RBC volume above the required minimum before proceeding to RBC depletion [34].

## Conclusions

We report positive outcomes for Spectra Optia BMC for RBC-depletion of bone marrow in clinical routine.

## Abbreviations

BM: bone marrow
CD: cluster of differentiation
RBC: red blood cell, erythrocyte
WBC: white blood cell, leukocyte
